# Supplementary material for: Structural insight into recognition of phosphorylated threonine‐4 of RNA polymerase II C‐terminal domain by Rtt103p
Source: EMBO Rep. 2017 May 3;18(6):906–13. doi: 10.15252/embr.201643723 (PMC5452035; doi:10.15252/embr.201643723)
Supplement: Supplementary file 1 — Expanded View Figures PDF [file EMBR-18-906-s001.pdf]

Expanded View Figures

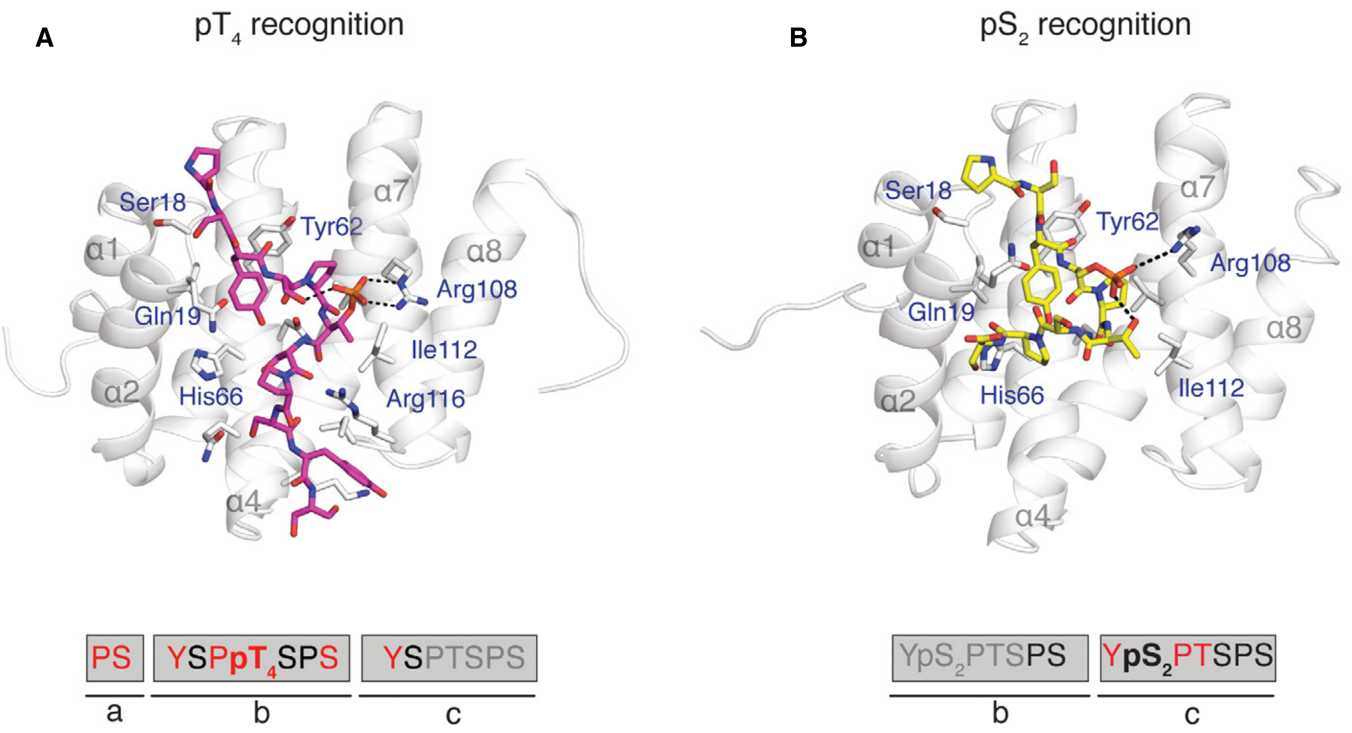

Figure EV1. Comparison of Rtt103p CID structures bound to differently phosphorylated CTD.

A, B Comparison of Rtt103p CID (grey helices) bound to the (A) pThr4-CTD (magenta sticks; PDB ID: 5LVF) or (B) to the pSer2-CTD peptide (yellow sticks; PDB ID: 2L0I.). Rtt103p CID residues involved in the interaction with CTD are shown in grey sticks and labelled with blue font. Sequences of peptides used in structure determination are indicated below the structures. Peptide residues shown on the image are highlighted in red and black font; the residues highlighted in red have intermolecular contacts used for the structure calculation; residues in grey are not displayed for clarity reasons or are missing coordinates.

Figure EV2. Rtt103p CID interacts with pSer2-CTD and pThr4-CTD using the same canonical interface.

In order to identify the surface of the CID involved in interaction with pThr4-CTD, we performed a $^{1}H^{15}N$-TROSY NMR titration experiment, where ^{15}N-labelled Rtt103 CID was titrated with pThr4 peptide. Titration confirmed that Rtt103p CID is interacting with pThr4-CTD peptide in an almost identical fashion as with pSer2-CTD, using the canonical surface of helices α2, α4 and α7 (Fig 1B and C). However, important rearrangements were observed for Val109 and Ile112, residues that lay in close proximity of P_{3b} and T_{4b}.

A Chemical shift perturbations (CSP) of the Rtt103p CID upon interaction with FAM-pSer2 CTD (red) or FAM-pThr4 CTD (grey) peptides plotted against residue number of Rtt103p CID. Secondary structure elements are shown below the *x*-axis. Helices involved in the interaction with phospho-peptides are coloured in black. FAM, 5,6-carboxyfluorescein.

B Overlay of ^{1}H-^{15}N TROSY spectra of complex of Rtt103p CID with FAM-pSer2 (red) and FAM-pThr4 (blue).

▶

© 2017 The Authors

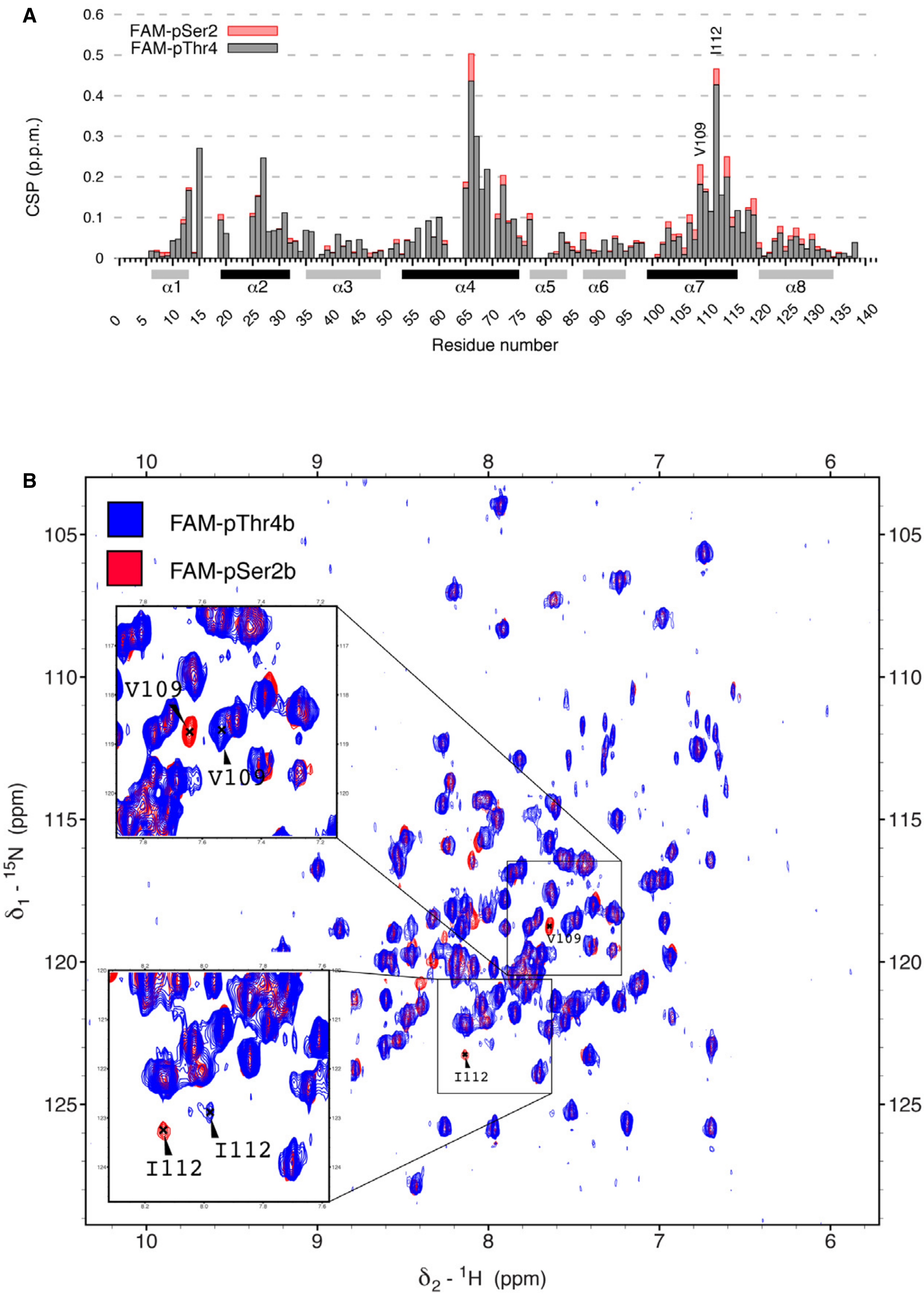

Figure EV2.

 © 2017 The Authors

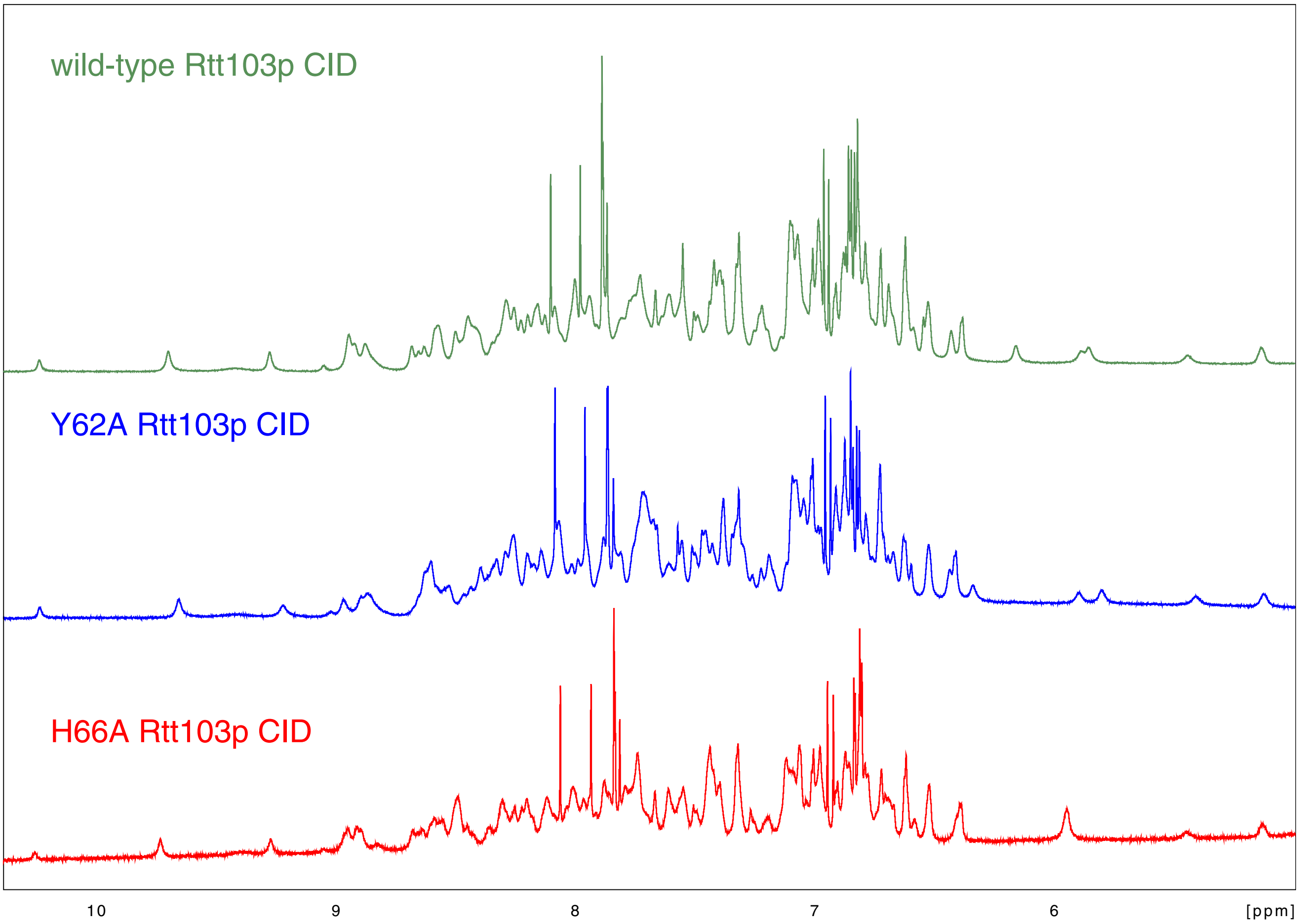

Figure EV3. Structural integrity of Rtt103p CID mutants.
Comparison of ^{1}H NMR spectra of the wild type (green), Y62A (blue) and H66A (red) mutants of Rtt103p CID; the region with NH backbone and side-chain resonances is shown. Data were collected on 850 MHz Bruker AVANCE III spectrometer at 293 K.

© 2017 The Authors

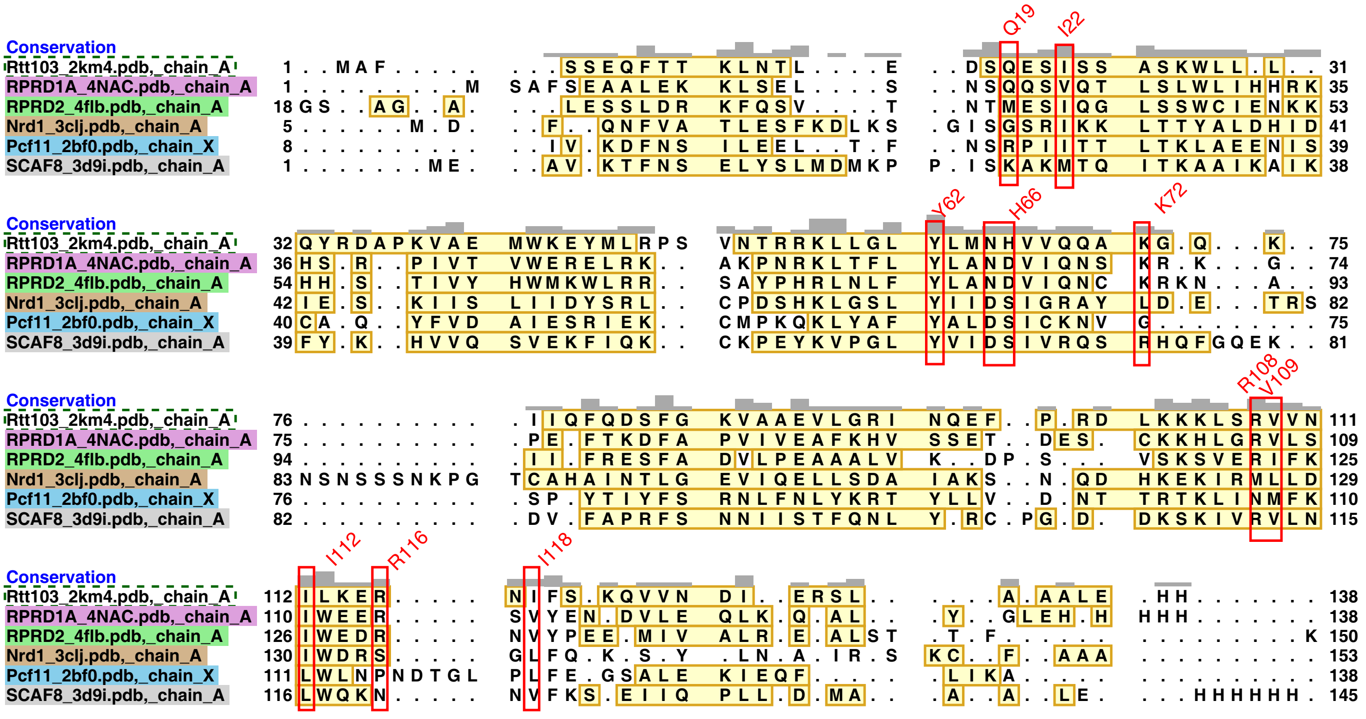

Figure EV4. Structural sequence alignment of CIDs.
Sequence alignment of CID based on superposition of the CID structures (PDB IDs: 2KM4, 4NAC, 4FLB, 3CLJ, 2BF0, 3D9I) using Align tool of UCSF Chimera [40]. In this type of alignment, residue types are not used, only their spatial proximities. Yellow boxes highlight structured elements; red boxes show key residues responsible for the CTD recognition according to numbering of Rtt103p.

Figure EV5. Intermolecular contacts between the Y_{1c} and Rtt103p CID.
Strip plots from 3D F1-$^{13}C/^{15}N$-filtered NOESY-[^{13}C-^{1}H]-HSQC showing intermolecular contacts between Y_{1c} and Arg116 (left) and Ile118 (middle, right).

 © 2017 The Authors

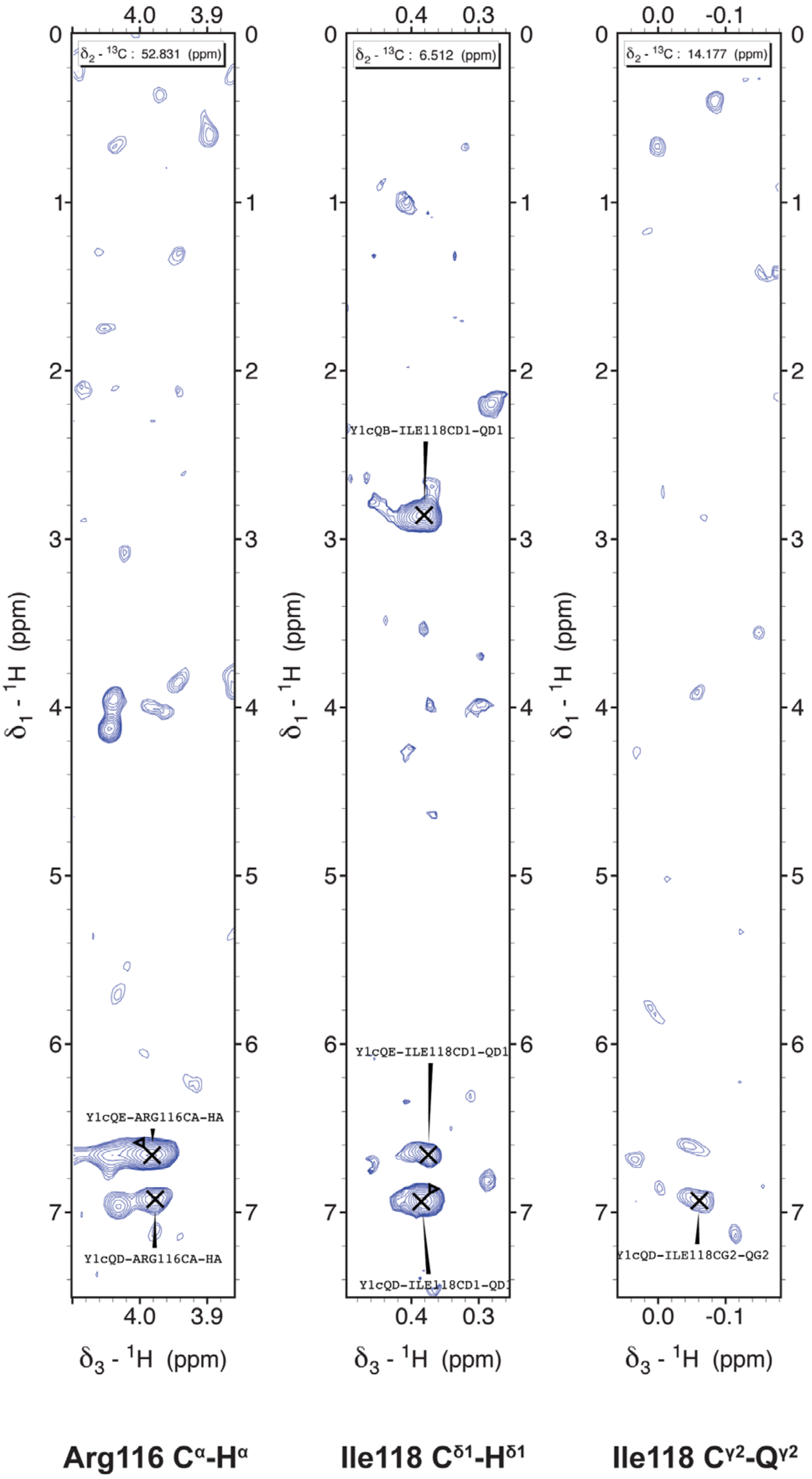
δ2 - 13C : 52.831 (ppm)
δ2 - 13C : 6.512 (ppm)
δ2 - 13C : 14.177 (ppm)
Y1cQB-ILE118CD1-QD1
Y1cQE-ILE118CD1-QD1
Y1cQD-ILE118CD1-QD1
Y1cQE-ARG116CA-HA
Y1cQD-ARG116CA-HA
Y1cQD-ILE118CG2-QG2
δ1 - 1H (ppm)
δ3 - 1H (ppm)
Arg116 Cα-Hα
Ile118 Cδ1-Hδ1
Ile118 Cγ2-Qγ2

Figure EV5.

Figure EV6. Double phosphorylation of CTD does not influence serine–proline peptide bond isomerization state population.

A Scheme of *cis*- and *trans*-isomers of X-proline peptide bond (X stands for any amino acid). Peptide bond is highlighted in blue.

B To establish conformational populations of mono- and di-phosphorylated peptides, [^{13}C,^{1}H] HSQC spectra of CTD peptides were measured. Comparison of [^{13}C,^{1}H] HSQC spectra of PSYS^{13C}PpTSPSYS (left) and PSYpS^{13C}PpTSPSYS (right). ^{1}H-^{13}C correlations of β and γ C-H pairs are shown. *Trans*-chemical shift region of Cγ/β highlighted in yellow [41].

C Overlay of [^{13}C,^{1}H] HSQC spectra from the NMR titration of the PSYS^{13C}PpTSPSYS peptide with non-labelled Rtt103p CID. Protein–peptide molar ratios for each titration step and corresponding colour of the spectrum are indicated on the right.

 © 2017 The Authors

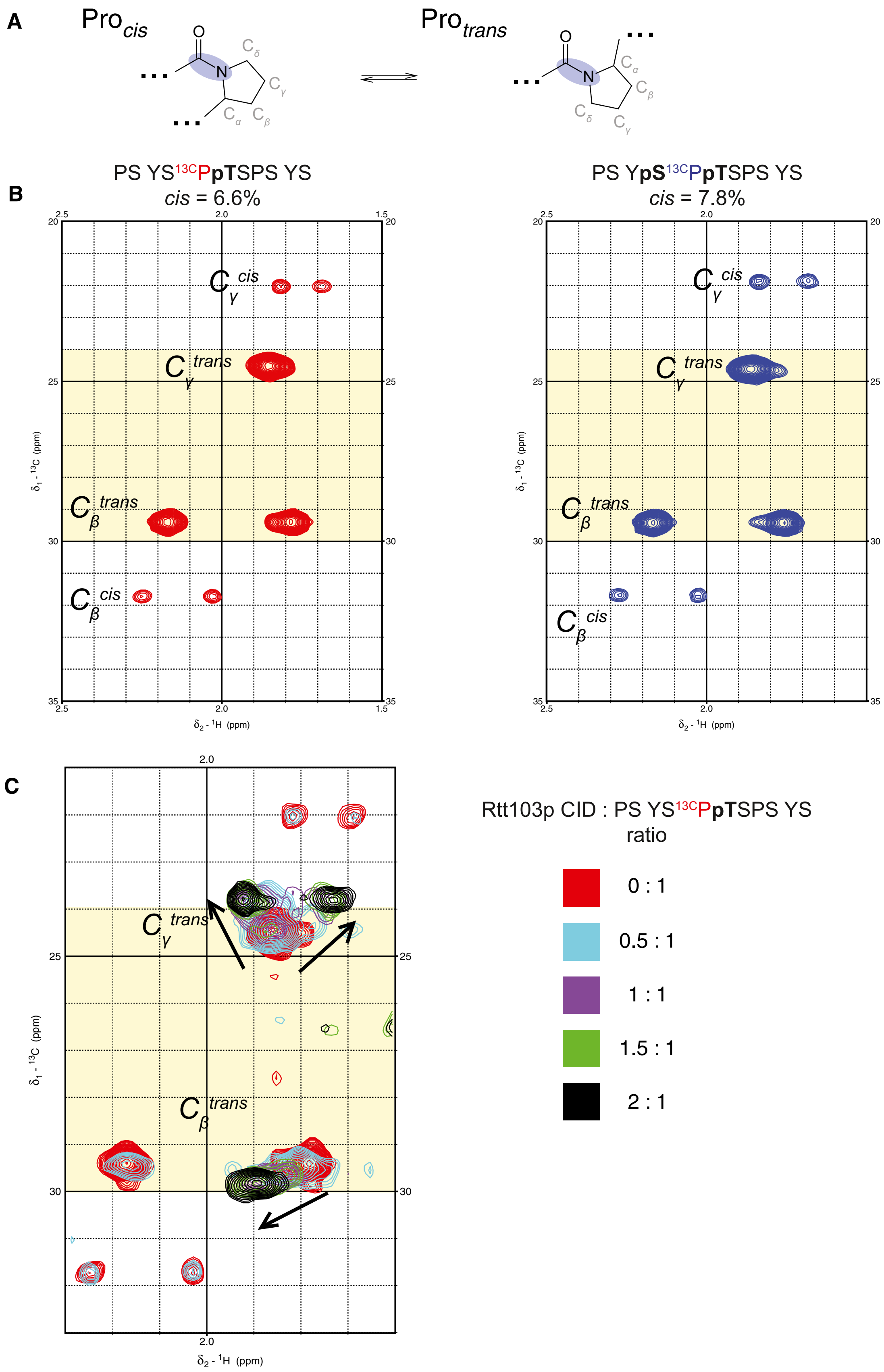
A
Pro cis
Pro trans
B
PS YS13CPpTSPS YS
cis = 6.6%
PS YpS13CPpTSPS YS
cis = 7.8%
δ1 - 13C (ppm)
δ2 - 1H (ppm)
C
Rtt103p CID : PS YS13CPpTSPS YS
ratio
0 : 1
0.5 : 1
1 : 1
1.5 : 1
2 : 1

Figure EV6.
